# Bidirectional two-sample Mendelian randomization analysis identifies causal associations between oxidative stress and Parkinson’s disease

**DOI:** 10.3389/fnagi.2024.1423773

**Published:** 2024-07-04

**Authors:** Lingling Lv, Zhe Long, Xuling Tan, Lixia Qin, Weiqian Yan, Hainan Zhang, Feng Ren, Chunyu Wang

**Affiliations:** ^1^Department of Neurology, The Second Xiangya Hospital, Central South University, Changsha, China; ^2^Department of Medical Genetics, The Second Xiangya Hospital, Central South University, Changsha, China; ^3^Key Laboratory of Hunan Province in Neurodegenerative Disorders, Central South University, Changsha, China; ^4^Department of Geriatric Surgery, The Second Xiangya Hospital, Central South University, Changsha, China

**Keywords:** Parkinson’s disease, oxidative stress, Mendelian randomization study, causal relationship, zinc

## Abstract

**Background:**

Observational studies have shown that oxidative stress (OS) is associated with Parkinson’s disease (PD). However, whether such observations reflect cause–effect remains largely unknown. To test this, we performed a two-sample bidirectional Mendelian randomization (MR) analysis to investigate the causal-effects between OS biomarkers and PD.

**Methods:**

We selected summary statistics data for single-nucleotide polymorphisms (SNPs) associated with catalase (*n* = 13), glutathione peroxidases (*n* = 12), superoxide dismutase (*n* = 13), vitamin A (*n* = 7), vitamin C (*n* = 10), vitamin E (*n* = 12), vitamin B12 (*n* = 8), folate (*n* = 14), copper (*n* = 6), Zinc (*n* = 7), and iron (*n* = 23) levels, and the corresponding data for PD from the International Parkinson Disease Genomics Consortium (IPDGC, 33,674 cases and 449,056 controls). Inverse-variance weighted (IVW) MR analyses were conducted to estimate associations of OS with PD. Reverse MR analysis was further performed to predict the causal effects of PD on the above OS biomarkers.

**Results:**

As for PD, the IVW method suggested that the Zinc (Zn) levels was significantly associated with PD (OR = 1.107, 95% CI 1.013–1.211; *p* = 0.025), which is consistent with results from the weighted median analyses. Moreover, the results remained consistent and robust in the sensitivity analysis. However, there were no significant associations of catalase, glutathione peroxidases, superoxide dismutase, vitamin A, vitamin C, vitamin E, vitamin B12, folate, copper, or iron with PD. As for OS, our reverse MR analysis also did not support a causal effect of liability to PD on OS.

**Conclusion:**

The MR study supported the causal effect of Zn on PD. These findings may inform prevention strategies and interventions directed toward OS and PD.

## 1 Introduction

Parkinson’s disease (PD), the most common neurodegenerative disease worldwide, is characterized by a progressive loss of substantia nigra (SN) dopaminergic neurons and accumulation of α-synuclein in the SN (Poewe et al., 2017). The main clinical manifestations of PD are motor symptoms (i.e., resting tremor, bradykinesia, rigidity, and postural instability) and nonmotor symptoms (i.e., depression, constipation, olfactory deficits, and cognitive impairment), which seriously affect the quality of life of patients and the economic burden of their family (Kalia and Lang, 2015). At present, the etiology and mechanism of PD remain elusive (Kalia and Lang, 2015). Consequently, the treatment for PD is primarily symptomatic, focusing on alleviating symptoms rather than slowing or halting disease progression (Bloem et al., 2021). Notably, with the discovery and studies of PD-related pathogenic genes (Blauwendraat et al., 2020), i.e., SNCA, PARKIN, PINK1, LRRK2, and DJ-1…, it has been revealed that oxidative stress (OS) is considered a key modulator in the occurrence and development of PD (Maiti et al., 2017). Mechanistically, OS refers to the overproduction of reactive oxygen species (ROS) and insufficient endogenous antioxidants, ultimately leading to cell dysfunction and death (Khan and Ali, 2018).

Substantial observational and experimental studies suggest that altered OS homeostasis may be involved in the etiology and pathogenesis of PD (Puspita et al., 2017; Chang and Chen, 2020; Jimenez-Moreno and Lane, 2020). It has been established that low levels of antioxidants, incapable of controlling ROS production, lead to neurodegeneration in PD (Medeiros et al., 2016). Oxidized lipids, proteins, and DNA can be seen in the substantia nigra of PD patients, which are all evidence of OS involvement (Abdelhamid and Nagano, 2023). LRRK2 mutant IPSC-derived DA neurons showed increased expression of genes involved in OS regulation and increased susceptibility to OS (Nguyen et al., 2011). Notably, studies have found that the concentration of oxidized proteins in the SN of healthy individuals is twice that of the caudate nucleus, putamen nucleus, and frontal cortex, suggesting a susceptibility of the SN to OS (Floor and Wetzel, 1998). These results suggest that OS plays an important role in PD.

Therefore, it is speculated that antioxidant treatment to regulate OS may be a strategy to treat PD. Although many antioxidants, such as vitamin E, vitamin C, and desferrioxamine, have been tested in clinical trials, none of them have been convincingly shown to improve neurodegeneration in PD patients (Fahn, 1992; Group, 1993, 1998; Devos et al., 2014). OS can be measured by the biomarkers of OS. Natural antioxidants are a very large diversified family of molecules classified by activity (enzymatic or nonenzymatic), and chemical structure (e.g., vitamins, trace elements, etc.). Catalase (CAT), glutathione peroxidases (GPx), superoxide dismutase (SOD), vitamin A, vitamin C, vitamin E, vitamin B12, folate, copper (Cu), zinc (Zn), and iron (Fe) are important antioxidants in the body. Impairments in antioxidant enzymes or non-enzymatic antioxidant networks, along with imbalance of redox-active metals, can induce the formation of toxic hydroxyl radicals and increase OS, leading to protein oxidation, misfolding, and ultimately cell death in PD (Umeno et al., 2017; Ben-Shushan and Miller, 2021). Furthermore, studies have also proven that many of the abovementioned antioxidants are altered in PD patients (Pichler et al., 2013; Medeiros et al., 2016; Anandhan et al., 2017), but not all studies agree with this view (Vinish et al., 2011). Moreover, it remains controversial as to whether such an OS injury is a cause or a downstream effect of PD. Observational research faces challenges in establishing causality regarding the causal relationship between OS and PD.

Mendelian randomization (MR) overcomes such confounders of observational studies by using genetic variants (SNPs) as instrumental variables (IVs) to infer the causal effect of an exposure on an outcome. MR can also reduce bias from reverse causation, because genetic phenotypes are postnatally unchanged through a lifetime. Therefore, in this study, we aimed to assess the bidirectional causal relationship between PD and OS. Understanding the role of OS in PD can lead to the development of targeted therapies aimed at mitigating its impact and potentially slowing down the progression of the disease.

## 2 Methods

### 2.1 Study design

**Figure 1:**
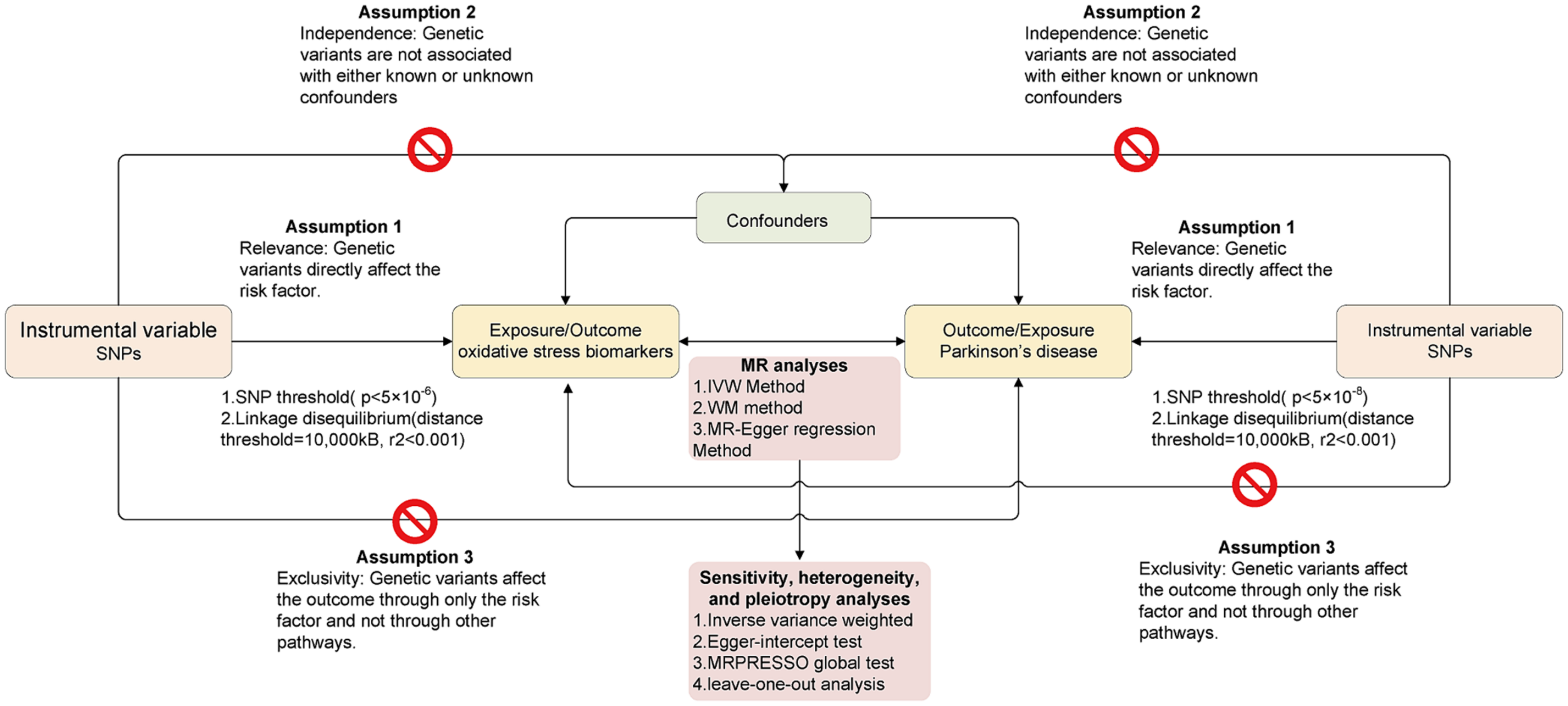
The overall flow chart of bidirectional MR study. The “
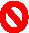
” means that genetic variants are not associated with confounders or cannot be directly involved in outcome but via the exposure pathway.

In this study, the bidirectional causal relationship between OS-related biomarkers and the risk of PD was explored using a bidirectional MR design. In the present research, we only extracted summarized data from the consortia. Ethical approval was not required because the study was based on existing publications and public databases. Figure 1 presents the MR analysis flow using the TwoSampleMR R package. To distinguish between a true negative result and a lack of validity of the MR studies, multiple sensitivity analyses were applied to ensure that the three MR assumptions were satisfied: (1) Relevance: The chosen genetic variants are associated with the exposure of interest. (2) Independence: The genetic variants are independent of confounding factors that might influence the exposure-outcome relationship. (3) Exclusivity: The genetic variants affect the outcome solely through their influence on the exposure, not through any alternative pathways.

### 2.2 GWAS data sources

We retrieved the genetic data of PD and OS biomarkers from the online platform of the Integrative Epidemiology Unit (IEU) open genome-wide association study (GWAS) project (https://gwas.mrcieu.ac.uk/, accessed on 20 March 2023). In this study, 11 biomarkers of OS were included, namely, CAT (Sun et al., 2018), G-Px (Sun et al., 2018), SOD (Sun et al., 2018), vitamin A (data were from the United Kingdom Biobank), vitamin C (Shin et al., 2014), vitamin E (data was from the United Kingdom Biobank), vitamin B12 (data were from the United Kingdom Biobank), folate (data were from the United Kingdom Biobank), Cu (Evans et al., 2013), Zn (Evans et al., 2013), and Fe (data were from the United Kingdom Biobank and Benyamin et al., 2014). The summary data of PD were obtained from a GWAS including 33,674 PD cases and 449,056 controls of the International Parkinson Disease Genomics Consortium (IPDGC) (Nalls et al., 2019). As mentioned, only the European population summarized data were adopted to avoid population heterogeneity. All datasets are publicly available online upon request. Detailed information on the GWAS datasets is described in Table 1.

**Table 1:** Detailed information regarding studies and datasets used in the present study.

Exposure or Outcome	Ancestry	Participants	Web source (accessed on 20 March 2023)	PMID	Journal	Ref
Oxidative stress						
Catalase (CAT)	European	3,301	https://gwas.mrcieu.ac.uk/datasets/prot-a-367/	29,875,488	Nature	Sun et al., 2018
Glutathione peroxidases (G-Px)	European	3,301	https://gwas.mrcieu.ac.uk/datasets/prot-a-1265/	29,875,488	Nature	Sun et al., 2018
Superoxide dismutase (SOD)	European	3,301	https://gwas.mrcieu.ac.uk/datasets/prot-a-2800/	29,875,488	Nature	Sun et al., 2018
Vitamin A	European	62,991	https://gwas.mrcieu.ac.uk/datasets/ukb-b-17406/	NA	NA	NA
Vitamin C	European	2,085	https://gwas.mrcieu.ac.uk/datasets/met-a-348/	24,816,252	Nature genetics	Shin et al., 2014
Vitamin E	European	64,979	https://gwas.mrcieu.ac.uk/datasets/ukb-b-6888/	NA	NA	NA
Vitamin B12	European	64,979	https://gwas.mrcieu.ac.uk/datasets/ukb-b-19524/	NA	NA	NA
Folate	European	64,979	https://gwas.mrcieu.ac.uk/datasets/ukb-b-11349/	NA	NA	NA
Copper	European	2,603	https://gwas.mrcieu.ac.uk/datasets/ieu-a-1073/	23,720,494	Human molecular genetics	Evans et al., 2013
Zinc	European	2,603	https://gwas.mrcieu.ac.uk/datasets/ieu-a-1079/	23,720,494	Human molecular genetics	Evans et al., 2013
Iron	European	64,979	https://gwas.mrcieu.ac.uk/datasets/ukb-b-20447/	NA	NA	NA
Iron	European	23,986	https://gwas.mrcieu.ac.uk/datasets/ieu-a-1049/	25,352,340	Nat Commun.	Benyamin et al., 2014
Disorder						
Parkinson Disease	European	33,674 cases and 449,056 controls	International Parkinson Disease Genomics Consortium (IPDGC). https://gwas.mrcieu.ac.uk/datasets/ieu-b-7/	31,701,892	Lancet Neurol	Nalls et al., 2019

### 2.3 Mendelian randomization

#### 2.3.1 Selection of IVs

We used the default settings in the R package TwoSampleMR, and extracted SNPs showing a significant association with each of the OS biomarkers at the conventional GWAS threshold (*p* < 5 × 10^−6^) for lack of significant SNPs. Similarly, IVs of PD were genome-wide significant SNPs (*p* < 5 × 10^−8^). To ensure the independence of IVs, all SNPs were clumped with a 10,000-kB window to a threshold of *r*^2^ < 0.001 to ascertain independence between genetic variants. Then, the proportion of variance of the exposures explained by the SNPs (*R*^2^) and *F*-statistics were calculated to estimate the strength of IVs to satisfy the first MR assumption (*F* > 10 for MR analyses; Palmer et al., 2011). The same approach was taken for the reverse MR; as such, we performed 22 bidirectional MR studies, where OS biomarkers and PD were regarded either as exposure or as the outcome.

#### 2.3.2 Mendelian randomization analyses

After clumping IVs, we first performed Steiger filtering to exclude SNPs explaining more variance in the outcome than in the exposure. Next, we harmonized the exposure and outcome data to produce data for MR analysis. In the main analysis, the inverse-variance weighted (IVW) method with random–effects was applied to combine the effect of different IVs. The IVW method is a widely used approach in MR because it provides a straightforward and interpretable summary estimate of the causal effect while leveraging genetic information. The odds ratio (OR) and 95% confidence intervals (CIs) were calculated for each SNP using IVW to assess the risk of exposure to outcome. In addition, we also performed the simple median method, weighted median (WM) method and MR–Egger regression method. The WM method is a median of the weighted estimates and provides a consistent effect even if 50% of IVs are pleiotropic. This means that even if a substantial proportion (up to 50%) of the IVs used in the analysis are pleiotropic, the WM method can still provide consistent and unbiased causal effect estimates. The MR–Egger regression method is used to detect possible violations of instrumental variable assumptions due to directional pleiotropy. It does this by allowing for an intercept term in the regression model, which accounts for any bias introduced by pleiotropic effects. Additionally, the Mendelian Randomization Pleiotropy RESidual Sum and Outlier (MRPRESSO) global test is used to detect outlier SNPs that may be biasing estimates through horizontal pleiotropy (i.e., SNPs with *p* < 0.05) and adjust for these. For heterogeneity, we applied Cochran’s Q statistic in the inverse variance weighting (IVW) and MR Egger regression methods. Finally, a leave-one-out analysis (LOO) was performed to detect whether any single SNP was disproportionately responsible for the result of each MR study. The results of the present study are shown as ORs (95% CIs) per genetically predicted increase in each lifestyle factor. For result visualization, we constructed scatter plots, forest diagrams, and funnel diagrams using the TwoSampleMR package and MRPRESSO package in the statistical program R. A *p* value less than 0.05 was considered statistically significant evidence of a causal association.

## 3 Results

### 3.1 Instrumental variables for Mendelian randomization

The number of IVs and the phenotypic variances they accounted for by the IVs are shown in Table 2. All of the variables were associated with OS at genome-wide significance. The variance in the exposures explained by their respective set of SNPs ranged from 0.2 to 20.1% (Tables 2, 3). Other than vitamin C, all instruments had an *F*-statistics of >21, which is above the standard cut-of (> 10) indicating sufficient instrumental strength (Bowden et al., 2018; Burgess et al., 2019).

**Table 2:** Results of the MR analyses between liability to oxidative stress and the PD risk.

Exposure	N SNPs	*r* ^2^	*F*-statistics	Inverse variance weighted			Weighted median			MR Egger		
				OR	CI	pval	OR	CI	pval	OR	CI	pval
CAT	13	0.107	27.478	1.015	0.923–1.116	0.765	1.031	0.909–1.168	0.638	0.962	0.716–1.292	0.801
G-Px	12	0.175	50.695	0.945	0.877–1.020	0.147	0.961	0.874–1.057	0.415	0.929	0.815–1.060	0.230
SOD	13	0.114	27.131	0.996	0.909–1.093	0.939	0.973	0.854–1.108	0.675	0.905	0.731–1.119	0.375
Vit. A	7	0.003	23.405	0.666	0.361–1.229	0.193	0.614	0.280–1.350	0.225	0.529	0.115–2.434	0.451
Vit. C	10	0.014	2.793	0.837	0.653–1.074	0.163	0.897	0.641–1.25	0.524	0.476	0.212–1.066	0.109
Vit. E	12	0.004	23.148	0.711	0.397–1.274	0.252	0.570	0.299–1.086	0.0875	0.611	0.121–3.097	0.565
Vit. B12	8	0.002	22.539	0.627	0.347–1.134	0.123	0.608	0.269–1.376	0.233	0.526	0.105–2.628	0.463
Folate	14	0.005	21.817	0.869	0.550–1.374	0.549	0.885	0.472–1.659	0.704	1.047	0.390–2.808	0.929
copper	6	0.080	35.180	1.039	0.925–1.168	0.517	1.096	0.970–1.238	0.140	0.865	0.711–1.052	0.219
zinc	7	0.100	33.106	1.107	1.013–1.211	0.025	1.136	1.003–1.286	0.044	1.064	0.753–1.504	0.739
Iron^1^	10	0.038	84.099	0.993	0.857–1.152	0.931	0.994	0.812–1.074	0.334	0.874	0.704–1.086	0.258
Iron^2^	13	0.005	22.575	0.721	0.464–1.119	0.144	0.700	0.380–1.290	0.252	0.402	0.122–1.325	0.162

*r*^2^ proportion of variance in exposure variable explained by SNPs; *F*-statistics, “strength” of the instrumental variable. ^1^GWAS data sources: https://gwas.mrcieu.ac.uk/datasets/ieu-a-1049/. ^2^GWAS data sources: https://gwas.mrcieu.ac.uk/datasets/ukb-b-20447/. CAT, Catalase; G-Px, Glutathione peroxidases; SOD, Superoxide dismutase; Vit. A, Vitamin A; Vit. C, Vitamin C; Vit. E, Vitamin E; Vit. B12, Vitamin B12.

**Table 3:** Associations between genetically predicted PD and oxidative stress biomarkers.

Outcome	N SNPs	*r* ^2^	*F*-statistics	Inverse variance weighted			Weighted median			MR Egger		
				OR	CI	pval	OR	CI	pval	OR	CI	pval
CAT	22	0.201	4475.823	0.951	0.881–1.025	0.190	0.949	0.854–1.054	0.331	1.017	0.848–1.221	0.854
G-Px	22	0.201	4475.823	1.024	0.944–1.110	0.571	1.007	0.899–1.128	0.902	1.121	0.923–1.361	0.264
SOD	22	0.201	4475.823	0.989	0.915–1.068	0.773	0.966	0.862–1.083	0.553	0.974	0.084–1.179	0.787
Vit. A	22	0.201	4475.823	0.992	0.974–1.010	0.405	0.987	0.962–1.013	0.314	0.990	0.945–1.038	0.690
Vit. C	15	0.120	3908.796	1.010	0.966–1.055	0.675	1.010	0.951–1.073	0.738	0.971	0.863–1.092	0.632
Vit. E	22	0.201	4475.823	0.988	0.967–1.009	0.264	0.995	0.968–1.022	0.696	1.008	0.954–1.065	0.780
Vit. B12	22	0.201	4475.823	0.998	0.977–1.020	0.857	0.989	0.965–1.015	0.411	0.977	0.926–1.031	0.413
Folate	22	0.201	4475.823	0.999	0.982–1.017	0.922	0.999	0.974–1.025	0.950	0.980	0.937–1.024	0.373
Copper	11	0.096	4265.144	0.908	0.799–1.032	0.141	0.885	0.748–1.048	0.156	0.881	0.645–1.204	0.448
Zinc	11	0.096	4265.144	1.034	0.931–1.170	0.600	0.988	0.832–1.174	0.893	0.931	0.689–1.260	0.657
Iron^1^	10	0.075	3650.035	1.015	0.965–1.068	0.562	1.022	0.957–1.092	0.610	1.079	0.903–1.290	0.426
Iron^2^	22	0.201	4475.823	1.002	0.985–1.019	0.813	1.002	0.979–1.026	0.849	0.978	0.937–1.021	0.321

*r*^2^ proportion of variance in exposure variable explained by SNPs; F-statistics, “strength” of the instrumental variable. ^1^GWAS data sources: https://gwas.mrcieu.ac.uk/datasets/ieu-a-1049/. ^2^GWAS data sources: https://gwas.mrcieu.ac.uk/datasets/ukb-b-20447/. CAT, Catalase; G-Px, Glutathione peroxidases; SOD, Superoxide dismutase; Vit. A, Vitamin A; Vit. C, Vitamin C; Vit. E, Vitamin E; Vit. B12, Vitamin B12.

### 3.2 Causal effect of oxidative stress biomarkers on PD

**Figure 2:**
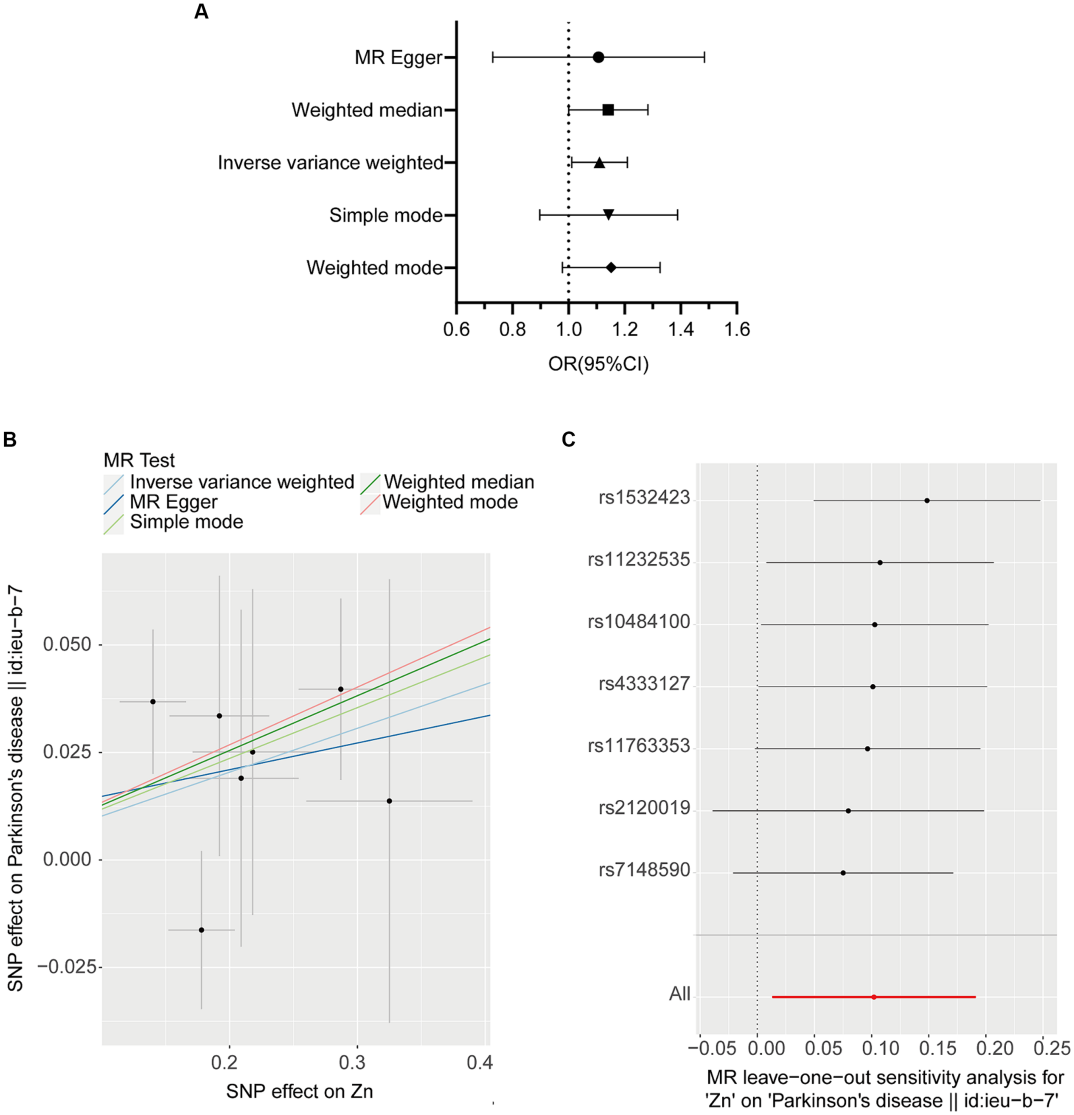
MR plots for the relationship of Zinc (*N* = 2,603) with PD (*N* = 482,730). **(A)** Forest plot of individual and combined SNP MR-estimated effect sizes. The effect estimates represent the odds that each S.D. increase in PD incidence increases odds of appendicular lean mass, and the error bars represent 95% CIs. **(B)** Scatter plot of SNP effects on Zinc vs. PD, with the slope of each line corresponding to the estimated MR effect per method. The *x*-axis represents the genetic association with Zinc risk; the *y*-axis represents the genetic association with the risk of PD. The data are expressed as raw β values with 95% Cis. **(C)** Leave one out plot, which detect outlier SNPs in Zinc and PD.

We investigated the association of OS with PD. The IVW method provided the results about the causal relationship between PD and 11 oxidative stress biomarkers. Figure 2 presents the forest plots of the associations between Zn and PD. The IVW method shows that genetically determined Zn was strongly associated with PD (OR = 1.107, 95% CI 1.013–1.211; *p* = 0.025), which is consistent with results from the WM method (OR = 1.136, 95% CI 1.003–1.286; *p* = 0.044) (Table 2; Figure 2). However, there were no significant associations of catalase, glutathione peroxidases, superoxide dismutase, vitamin A, vitamin C, vitamin E, vitamin B12, folate, copper, or iron with PD (Table 2).

### 3.3 Sensitivity analysis

No heterogeneity was detected by MR–Egger and IVW tests (Supplementary Table S1). Additionally, no evidence of between-SNP directional and horizontal pleiotropy was detected by the MR-PRESSO global test and MR–Egger regression (Supplementary Table S1). We further performed a leave-one-out analysis to detect whether the causal estimate was driven by any single SNP, which revealed a consistent inverse association between genetically predicted Zn levels and the risk of PD (Figure 2C).

### 3.4 Causal effect of PD on oxidative stress biomarkers

Additionally, reverse MR analysis was conducted to investigate the potential causal effects of PD on 11 OS biomarkers. In these studies, we used 10–22 PD-associated SNPs, which explained 0.075–0.201 of the variances in PD risk and had an *F*-statistic of more than 3,650. The IVW test results showed no statistically significant associations of PD with OS. The null findings were supported by other MR methods (Table 3). The Egger intercept did not identify any pleiotropic SNP (*p* of Egger intercept from 0.327 to 0.930). Moreover, the MR-PRESSO global test did not identify outlier SNPs. Significant heterogeneity was apparent in our IVs for PD (only when Vitamin E and Vitamin B12 is the outcome) (IVW and MR Egger, *Q* pvalue < 0.1) (Supplementary Table S2). Similarly, there was no apparent sign of significant heterogeneity as assessed by leave-one-out analysis.

## 4 Discussion

This study represents the inaugural bidirectional investigation into the potential involvement of OS in PD. Our research delves into the causal connections between 11 OS biomarkers and the risk of PD, offering fresh perspectives on the correlation between OS status and PD. Notably, among the 11 OS biomarkers studied, we discovered genetic evidence supporting the causal link between Zn and PD. Our findings exhibited considerable robustness across various MR methods, each built upon different assumptions regarding horizontal pleiotropy. These findings may inform prevention strategies and interventions directed toward OS and PD.

Although many observational studies have highlighted the link between OS and PD, there have been few relevant MR studies. These MR studies usually focus on iron. Only one one-way MR study that examined causality between serum iron levels and PD suggested a causal relationship between elevated iron levels (instrumental variables, HFE rs1800562, HFE rs1799945, and TMPRSS6 rs855791) and a reduced risk of PD (OR 0.88, 95% CI 0.82–0.95; *p* = 0.001; Pichler et al., 2013). However, in our study, we did not find a causal relationship between iron levels and PD.

For other trace elements, suggestive causal associations were found between Zn and PD risk in this work (OR = 1.107, CI = 1.013–1.211, *p* = 0.025). In our previous study, they showed that alterations in zinc homeostasis have long been implicated in PD. Zinc exposure has been identified as an environmental risk factor for PD, and PD patients were clearly shown to have higher zinc exposure than those without PD (OR = 11.6, 95% CI: 1.51–90.90) (Pals et al., 2003). In addition, postmortem of idiopathic PD patients and PD mouse models showed Zn^2+^ depositions in dopaminergic neurons (Wang et al., 2023). Zn^2+^ chelation attenuated the loss of nigrostriatal dopaminergic neurons and the associated motor deficits induced by 6-OHDA or paraquat. The evidence suggests that endogenous Zn^2+^ plays a key role in the pathophysiology of PD. Zinc homeostasis (balance) is important for OS. On the one hand, Zn^2+^ can influence the activity of antioxidant enzymes and signaling pathways. On the other hand, overload of intracellular Zn^2+^ can lead to OS (Sikora and Ouagazzal, 2021). However, previous studies have found that lower serum and plasma zinc levels are associated with a higher risk of PD (Finkelstein et al., 2013; Du et al., 2017), which contradicts our findings. This discrepancy may stem from differences in study design and population sample sizes. Our study utilizes a robust genetic approach, distinct from the observational and experimental designs employed in other research. Genetic evidence offers a more direct assessment of causal relationships, thereby reducing potential confounding factors and biases. Additionally, variations in the populations studied, including differences in genetic backgrounds, environmental exposures, and dietary habits, might influence the results. Further research is needed to elucidate the specific mechanisms by which Zn affects PD and to identify potential therapeutic targets for intervention. Cu^2+^ is another common redox-active metal in addition to Zn and iron, but in this study, the causal effect of Cu^2+^ on PD was not significant. Redox-active metals are common cofactors that promote amyloid aggregation in neurodegenerative diseases, such as PD (Ben-Shushan and Miller, 2021). Overall, we speculate that redox-active metals do not entirely mediate the occurrence of PD through OS.

Enzymatic antioxidants, such as GPx, CAT, and SOD, are a critical component of the body’s defense mechanisms against OS (Emamzadeh and Surguchov, 2018). It could inhibit the formation of peroxide and remove free radicals. These enzymatic antioxidants work in concert to maintain the delicate balance between ROS production and neutralization, helping to prevent oxidative damage and maintain cellular health. Studies found that there were lower levels of antioxidant activity of SOD, CAT, and GPx in the PD group compared to controls (Khan and Ali, 2018; Duarte-Jurado et al., 2021). For example, decreased levels of GPx in the substantia nigra pars compacta (SNpc) are one of the earliest biochemical changes observed in PD (Pearce et al., 1997; Tobón-Velasco et al., 2010; Smeyne and Smeyne, 2013). GPx is an important antioxidant enzyme that helps protect cells from oxidative stress by neutralizing harmful ROS and peroxides. One of the biochemical alterations that has been detected in the postmortem brains of PD patients is the selective GPx reduction in the SNpc. *In vivo* and *in vitro* experiments have shown that replenishing intracellular GPx levels can prevent oxidative damage and maintain mitochondrial function in dopaminergic cells (Smeyne and Smeyne, 2013; Duarte-Jurado et al., 2021). However, these findings cannot resolve the previous debate on whether these enzymatic antioxidants are the cause of PD or a consequence of it. To date, there are no other MR studies that have assessed the causal association of enzymatic antioxidants and PD risk. In our MR study, it appeared likely that there is no causality between enzymatic antioxidants and PD risk.

Several studies have identified an association of nonenzymatic antioxidants, vitamins (vitamin A, E, C, B12, and folate) with PD (Hughes et al., 2016). Low plasma levels of exogenous antioxidants (including vitamin C) have been reported in patients with neurodegenerative diseases such as PD. In addition, a higher prevalence of PD with subclinical vitamin C deficiencies has also been reported (Duarte-Jurado et al., 2021). It has been found that vitamin E protects dopaminergic neurons against MPTP-mediated toxicity. A cross-sectional study reported that vitamin E status was inversely associated with PD risk (Miyake et al., 2011). However, our results do not support the hypothesis that vitamins substantially affect the risk of PD, which is in line with other case–control studies (Logroscino et al., 1996; Scheider et al., 1997; Powers et al., 2003). Furthermore, when antioxidant vitamins were tested in clinical trials, their effects were not reproduced. A prospective cohort study found that the intake of antioxidant vitamins does not reduce the risk of PD (Hughes et al., 2016). Our updated results suggest that causality causation is unlikely. Therefore, these results support the hypothesis that antioxidant supplementation is unlikely to be of clinical benefit in the prevention of PD. It is important to note that our null findings do not invalidate the role of oxidative stress in PD. Larger epidemiological studies and directed laboratory studies are needed to determine the biochemical and chemical biological bases for these associations.

The advantages of the present study include the following aspects. First, 11 OS biomarkers were included, which made it the most comprehensive MR study between PD and the OS system. Second, we used several essential methods to verify the accuracy of the results, such as calculating the F statistic and power, and conducting heterogeneity and pleiotropy tests. There were some limitations as well. First, in order to extract more SNPs showing a significant association with OS biomarkers to maintain the study power in MR, the threshold of the *p* value was set as *p* < 5 × 10^−6^, meaning that the proportion of variance explained for the associations between some IVs and OS biomarkers was relatively small. Additionally, this is a European based study, and our findings cannot be generalized to other populations.

## 5 Conclusion

In conclusion, this study used large exposure and outcome GWASs to conduct MR analysis to infer a causal relationship between OS and PD. We found robust genetic evidence for an association between Zn levels and higher PD risk, and the other antioxidants do not affect the risk of PD. This finding contrasts with preclinical studies, where antioxidants play a significant role in maintaining neuronal survival and activity in PD models (Duarte-Jurado et al., 2021). However, such effects have not been observed in clinical trials, as antioxidants have failed to modify the disease in terms of clinical symptoms or the onset of PD (Logroscino et al., 1996). This has given rise to the hypothesis that circulating antioxidant levels might not be representative of antioxidant capacity, and that increasing the levels of antioxidant in blood (either by nutritional intake or supplements) does not necessarily result in additional antioxidative effects. Our study offers novel insights into the complex interplay between antioxidant status and PD.

## Data availability statement

The original contributions presented in the study are included in the article/Supplementary material; further inquiries can be directed to the corresponding authors.

## Author contributions

LL: Writing – original draft, Data curation, Formal analysis, Visualization. ZL: Writing – review & editing, Data curation. XT: Writing – review & editing. LQ: Writing – review & editing, Funding acquisition. WY: Writing – review & editing, Funding acquisition. HZ: Writing – review & editing. FR: Writing – review & editing, Methodology. CW: Writing – review & editing, Conceptualization.

## Glossary

CAT: Catalase
CIs: Confidence intervals
Cu: Copper
Fe: Iron
GPx: Glutathione peroxidases
GWAS: Genome-wide association study
IVs: Instrumental variables
IVW: Inverse-variance weighted
IPDGC: International Parkinson Disease Genomics Consortium
MR: Mendelian randomization
MR-PRESSO: Mendelian Randomization Pleiotropy RESidual Sum and Outlier
OS: Oxidative stress
OR: Odds ratio
PD: Parkinson’s disease
ROS: Reactive oxygen species
SNPs: Single-nucleotide polymorphisms
SN: Substantia nigra
SNpc: Substantia nigra pars compacta
SOD: Superoxide dismutase
WM: Weighted median
Zn: Zinc
