# Supplementary material for: Bidirectional two-sample Mendelian randomization analysis identifies causal associations between oxidative stress and Parkinson’s disease
Source: Front Aging Neurosci. 2024 Jul 4;16:1423773. doi: 10.3389/fnagi.2024.1423773 (PMC11254677; doi:10.3389/fnagi.2024.1423773)
Supplement: Supplementary file 1 [file Table_1.docx]

Table S1. Sensitivity analysis, heterogeneity, and pleiotropy, investigating MR assumption violation

| Exposure | Heterogeneity tests | | | | | | | | | | Test for directional and horizontal pleiotropy | | | | | | | | | |
| --- | --- | --- | --- | --- | --- | --- | --- | --- | --- | --- | --- | --- | --- | --- | --- | --- | --- | --- | --- | --- |
|  | MR Egger | | | | Inverse variance weighted | | | | | | MR-PRESSO global | | | | | MR Egger | | | |  |
|  | Q | Q_df | Q_pval | | Q | | Q_df | | Q_pval | | | RSSobs | | | pval | | egger_intercept | se | pval | |
| CAT | 6.448 | 11 | | 0.842 | | 6.588 | | 12 | | 0.884 | | | 7.821 | | 0.879 | | 0.009 | 0.025 | 0.715 | |
| G-Px | 4.264 | 10 | | 0.935 | | 4.365 | | 11 | | 0.958 | | | 5.196 | | 0.965 | | 0.005 | 0.016 | 0.758 | |
| SOD | 10.753 | 11 | | 0.464 | | 11.732 | | 12 | | 0.467 | | | 13.850 | | 0.526 | | 0.021 | 0.021 | 0.344 | |
| Vit. A | 2.932 | 5 | | 0.711 | | 3.035 | | 6 | | 0.804 | | | 4.044 | 0.802 | | | 0.010 | 0.032 | 0.760 | |
| Vit. C | 7.188 | 8 | | 0.517 | | 9.265 | | 9 | | 0.413 | | | 11.458 | 0.418 | | | 0.040 | 0.028 | 0.187 | |
| Vit. E | 18.762 | 10 | | 0.043 | | 18.835 | | 11 | | 0.064 | | | 21.838 | 0.082 | | | 0.007 | 0.033 | 0.848 | |
| Vit. B12 | 6.048 | 6 | | 0.418 | | 6.102 | | 7 | | 0.528 | | | 7.839 | 0.586 | | | 0.007 | 0.031 | 0.825 | |
| Folate | 9.500 | 12 | | 0.660 | | 9.673 | | 13 | | 0.720 | | | 11.295 | 0.718 | | | -0.009 | 0.021 | 0.685 | |
| Cu | 3.697 | 4 | | 0.449 | | 8.065 | | 5 | | 0.153 | | | 12.438 | 0.22 | | | 0.046 | 0.022 | 0.105 | |
| Zn | 5.817 | 5 | | 0.324 | | 5.881 | | 6 | | 0.437 | | | 12.481 | 0.246 | | | 0.009 | 0.036 | 0.823 | |
| Iron^1^ | 11.013 | 8 | | 0.200 | | 14.142 | | 9 | | 0.117 | | | 17.500 | 0.194 | | | 0.019 | 0.0124 | 0.170 | |
| Iron ^2^ | 8.541 | 11 | | 0.664 | | 9.608 | | 12 | | 0.650 | | | 11.437 | 0.645 | | | 0.021 | 0.021 | 0.324 | |

^1^GWAS data sources: <https://gwas.mrcieu.ac.uk/datasets/ieu-a-1049/>

^2^GWAS data sources: <https://gwas.mrcieu.ac.uk/datasets/ukb-b-20447/>

CAT, catalase; G-Px, glutathione peroxidases; SOD, superoxide dismutase; Vit. A, vitamin A; Vit. C, vitamin C; Vit. E, vitamin E; Vit. B12, vitamin B12;

Table S2. Sensitivity analysis, heterogeneity, and pleiotropy, investigating reverse MR assumption violation

| Outcome | Heterogeneity tests | | | | | | | | | | Test for directional horizontal pleiotropy | | | | | | | | |
| --- | --- | --- | --- | --- | --- | --- | --- | --- | --- | --- | --- | --- | --- | --- | --- | --- | --- | --- | --- |
|  | MR Egger | | | | Inverse variance weighted | | | | | | MR-PRESSO global | | | | MR Egger | | | |  |
|  | Q | Q_df | Q_pval | | Q | | Q_df | | Q_pval | | | RSSobs | | pval | | egger_intercept | se | pval | |
| CAT | 19.346 | 20.000 | | 0.499 | | 19.989 | | 21.000 | | 0.522 | | | 21.410 | 0.551 | | -0.012 | 0.016 | 0.432 | |
| G-Px | 22.671 | 20 | | 0.305 | | 23.816 | | 21 | | 0.302 | | | 26.867 | 0.326 | | -0.017 | 0.017 | 0.327 | |
| SOD | 22.038 | 20 | | 0.338 | | 22.071 | | 21 | | 0.395 | | | 24.751 | 0.463 | | 0.003 | 0.016 | 0.865 | |
| Vit. A | 22.519 | 20 | | 0.313 | | 22.528 | | 21 | | 0.370 | | | 24.419 | 0.432 | | 0.0003 | 0.004 | 0.930 | |
| Vit. C | 8.518 | 13 | | 0.808 | | 9.006 | | 14 | | 0.831 | | | 10.230 | 0.874 | | 0.006 | 0.009 | 0.497 | |
| Vit. E | 31.709 | 20 | | 0.047 | | 32.691 | | 21 | | 0.050 | | | 36.282 | 0.057 | | -0.004 | 0.005 | 0.440 | |
| Vit. B12 | 31.045 | 20 | | 0.055 | | 32.122 | | 21 | | 0.057 | | | 36.532 | 0.065 | | 0.004 | 0.004 | 0.415 | |
| Folate | 21.297 | 20 | | 0.380 | | 22.258 | | 21 | | 0.385 | | | 25.748 | 0.377 | | 0.004 | 0.004 | 0.353 | |
| Cu | 4.668 | 9 | | 0.862 | | 4.712 | | 10 | | 0.910 | | | 14.900 | 0.528 | | 0.005 | 0.024 | 0.840 | |
| Zn | 8.240 | 9 | | 0.510 | | 8.785 | | 10 | | 0.553 | | | 17.171 | 0.401 | | 0.017 | 0.023 | 0.479 | |
| Iron^1^ | 3.149 | 8 | | 0.925 | | 3.642 | | 9 | | 0.933 | | | 9.472 | 0.836 | | -0.009 | 0.013 | 0.503 | |
| Iron^2^ | 19.574 | 20 | | 0.485 | | 21.041 | | 21 | | 0.456 | | | 22.874 | 0.547 | | 0.004 | 0.004 | 0.240 | |

^1^GWAS data sources: <https://gwas.mrcieu.ac.uk/datasets/ieu-a-1049/>

^2^GWAS data sources: <https://gwas.mrcieu.ac.uk/datasets/ukb-b-20447/>

CAT, catalase; G-Px, glutathione peroxidases; SOD, superoxide dismutase; Vit. A, vitamin A; Vit. C, vitamin C; Vit. E, vitamin E; Vit. B12, vitamin B12;
